# Cosmetic Outcomes of the First Bodybuilder Using a Low-Cost Modified Culture Technique for Burn Wound Coverage: A Case Report and Long-Term Follow-Up

**DOI:** 10.3390/ebj6020029

**Published:** 2025-06-03

**Authors:** Wayne George Kleintjes, Tarryn Kay Prinsloo

**Affiliations:** 1 Department of Surgical Sciences, Faculty of Medicine and Health Sciences, Stellenbosch University Medical School, Francie van Zijl Avenue, Parow, Cape Town 7505, South Africa; 2 Western Cape Provincial Adult Tertiary Burns Centre, Tygerberg Hospital, Tygerberg, Francie van Zijl Avenue, Parow, Cape Town 7505, South Africa; 3 Department of Biomedical Sciences, Faculty of Health and Wellness Sciences, Cape Peninsula University of Technology, Bellville, Cape Town 7535, South Africa; 4 Department of Emergency Medical Sciences, Faculty of Health and Wellness Sciences, Cape Peninsula University of Technology, Bellville, Cape Town 7535, South Africa

**Keywords:** burn, cultured epidermal autografts, body builder, cosmesis, skin graft, graft take, Tygerberg hospital, skin transplantation

## Abstract

Cultured epidermal autografts (CEAs) serve as an alternative permanent skin replacement, though high costs often limit their use in resource-constrained settings and to life-saving cases. This case report presents the first documented cosmetic application of a modified CEA technique in a bodybuilder, demonstrating favorable aesthetic outcomes. A 28-year-old Black male with a 20% total body surface area burn sustained in a domestic fire exhibited superficial and deep partial-thickness burns to the face, arms, torso, and feet. Refusing grafts from visible donor sites, treatment using a low-cost modified CEA approach was employed to minimize donor site morbidity. Keratinocytes harvested from a groin biopsy were cultured on Cutimed Sorbact^®^ (Essity AB, BSN Medical (Pty) Ltd., Pinetown, RSA) dressings with autogenous plasma and hydrogel supplementation and incubated at 37 °C for two weeks. Xenografts provided temporary coverage before CEA transplantation. Graft take was 85%, with minor (15%) loss at 21 days, requiring small autograft coverage. At two months, the Vancouver Scar Scale score was 4, indicating optimal pigmentation, smoother texture, and minimal scarring. These findings align with limited studies on CEAs for cosmetic applications, suggesting this cost-effective technique may broaden the scope of CEAs beyond life-saving interventions to include aesthetic reconstruction, reducing both donor site morbidity and scarring.

## 1. Introduction

Traditional skin autografts, including both full- and split-thickness grafts (STSG), remain the “gold standard” for burn wound coverage [1,2] due to their reduced risk of complications commonly associated with heterologous or synthetic transplants. These grafts, therefore, allow for permanent wound closure without eliciting immune rejection, making them the reliable therapeutic modality in burn care. However, the graft-to-wound size ratio is not always favorable [3], particularly in patients with extensive burn injuries, where the availability of suitable donor sites is often limited or insufficient [4,5]. Additionally, repeated harvesting increases morbidity risk and requires a healing period [6,7]. In clinical scenarios where donor site limitations pose a significant challenge, cultured epidermal autografts (CEAs) have emerged as a promising alternative for permanent wound closure. These grafts can be expanded in vitro from a small biopsy and favorable outcomes have been reported when used in conjunction with allografts or xenografts for temporary coverage before transplantation (composite technique) [8,9]. Despite its potential, its inclusion into standard routine procedure has been met with apprehension due to a number of logistical and clinical limitations such a fragile handling, long-term coverage, susceptibility to infection, and high failure rates [10,11,12]. However, the prohibitive cost of conventional CEA methods [10] is perhaps the main limitation affecting resource-constraint clinical settings such as those in low- and middle-income countries. The Western Cape Provincial Adult Tertiary Burn Centre (WCPATBC) at Tygerberg Hospital in Cape Town, South Africa, operates under such constraints. In order to mitigate some of the limitations, a cost-effective CEA technique was developed at the WCPATBC, which utilizes readily available materials—such as standard gauze and paediatric incubators—to cultivate CEA, thereby eliminating the need for specialized laboratory infrastructure [13]. Notably, patients that received these CEA transplants had a graft-take rate [8] that exceeded those reported in the largest CEA case series study [9], to date. While primarily used for severe burns with poor survival outcomes attributed to severe burn injuries and the absence of available donor sites, this technique was also applied to non-life-threatening cases, suggesting its broader utility. Of particular interest is the potential application in improving aesthetic outcomes, which comes as no surprise since improved scar quality and overall aesthetic outcome following CEA treatment have been reported [4,10,14,15], thereby reinforcing its value beyond survival-oriented care. Given that previous publications on this modified technique focused on life-saving applications, with graft-take and success rates as primary outcomes, this report demonstrates its versatility through its successful cosmetic application in a bodybuilder’s burn injury treatment.

## 2. Detailed Case Description

### 2.1. Case Presentation

**Figure 1:**
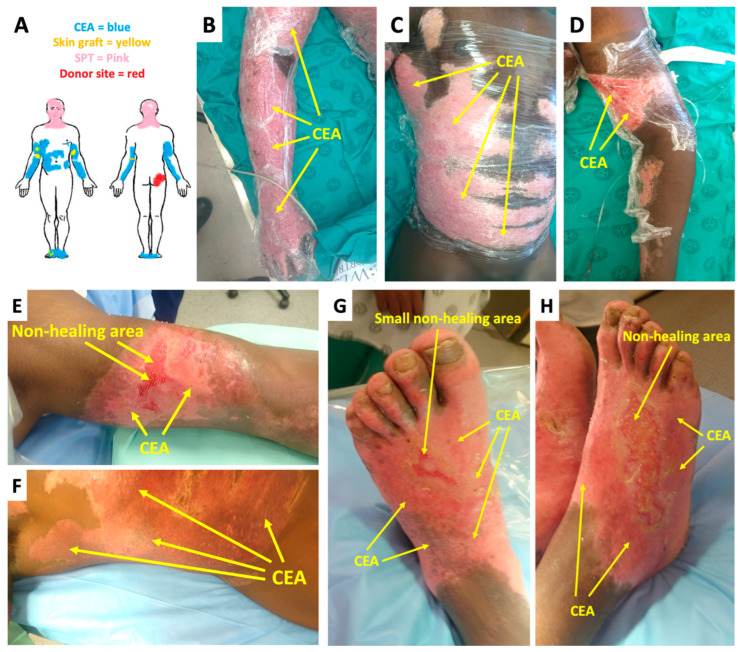
CEA and autograft after transplant: (**A**) Donor site and grafting layout; (**B**–**D**) CEA appearance 7 days after transplant; (**E**–**H**) CEA appearance 21 days after transplant during final graft take assessment (CEAs—cultured epidermal autografts, SPT—superficial partial thickness).

A 28-year-old Black male sustained 20% total body surface area (TBSA) burns from an accidental domestic fire, with an abbreviated burn severity index score of 5. Superficial partial-thickness burns affected the face and neck, while deep partial-thickness burns involved the medial and anterior arms, right torso, and dorsal feet up to the ankles (Figure 1A). He had no inhalation injury, comorbidities, or chronic medication use. Initially treated (first aid cleaning and dressing) at a trauma center, he was referred to WCPATBC, where standard wound care (cleaning and Cutimed Sorbact^®^ hydrophobic dressing [Essity AB, BSN Medical (Pty) Ltd., Pinetown, RSA]) and fluid resuscitation (modified Parkland^TM^ formula) were initiated. No primary grafting was performed during the first week post admission. Due to the patient’s profession as a bodybuilder, he declined graft harvesting from the scalp or other visible donor sites. Consequently, CEAs were prepared from a skin biopsy and applied in the second week, after evidence of secondary intention healing was observed and to minimize donor site morbidity. He remained hospitalized for four weeks and attended follow-up clinic visits at 2 weeks and 2 months post discharge.

### 2.2. Treatment and Outcomes

A detailed culture protocol has been previously described [13]. Within the WCPATBC, a seminar room (4 × 3.5 m) was repurposed into a laboratory space, and two donated pediatric incubators were installed for cell culture use. This location was selected for its proximity to the operating theatre, allowing for the efficient transport of skin biopsies and, subsequently, the CEAs intended for grafting. The incubators were selected for their capacity to maintain a controlled, sterile environment essential for cell culture. Both internal and external surfaces were disinfected using chlorhexidine, followed by sterile, alcohol gauze wipes. The temperature of each incubator was set and maintained at 37 °C, with daily humidification achieved by adding sterile water into the external inlet. On day 1 of culturing in the operating room, a 4 × 2 cm skin biopsy from the left inguinal region was collected and transferred to the “laboratory” for preparation on a sterile surface. The epidermis was separated from the dermis using a 15/0 scalpel, further fragmented by carving, and then submerged in a sterile water–trypsin solution for 2 h. A Recell^®^ kit sieve (Avita Medical, Perth, Australia) was used to filter the trypsin, and the remaining epithelial cells (specs) were transferred to two growth substrates: (1) Cutimed Sorbact^®^ dressing (Essity AB, BSN Medical (Pty) Ltd., Pinetown, RSA) with platelet-rich plasma (PRP) and (2) PRP-containing specimen bottles for suspension growth. Both were placed into the sterile pediatric incubator for cell cultivation. The dressing-based substrate was supplemented daily with fresh PRP and every third day with CutimedGel hydrogel (Cutimed, Essity Medical Solutions) to prevent desiccation [13]. The fresh PRP was prepared daily by withdrawing 6 mL of blood from existing intravenous lines into acid-citrate-dextrose tubes. The tubes were then centrifuged at 3500 rpm for 8 min. The resulting supernatant plasma was carefully aspirated and applied to both growth substrates in the incubators: the dressing containing the epithelial cells and the suspension bottles. Supplementation continued until confluence was reached at 2 weeks, which was confirmed macroscopically by the thicker, gelatinous appearance, dressing shrinkage (indicative of fibroblast activity), and a color change from pale white/grey to brown (suggesting pigmentation). Previous microscopic findings confirmed the differentiated cellular nature at 2 weeks resembling that of native skin, supporting both the adequacy of the shortened culture period and the graft suitability for clinical wound coverage [16]. From biopsy retrieval (day 1) and for the duration of the culture period, wounds were temporarily covered with commercially available xenografts (EZ Derm^®^, Molnlycke Health Care, UC LLC, Norcross, GA, USA) until CEA reached confluency (2 weeks) and was ready for transplantation. The xenograft’s superficial layers were removed using the Versajet^TM^ hydro-debridement system (Smith+Nephew, Inc., Fort Worth, TX, USA), and hemostasis was achieved using swabs saturated in an adrenaline solution comprising 1 L Ringer’s lactate mixed with one ampoule of adrenaline and 800 mg of lignocaine. Following debridement, the CEA-containing dressing was then directly transplanted onto prepared wound beds [9,13]. Graft take was assessed at days 7 and 21 (final assessment) and at 2 months. The overall quality of the CEA transplant and donor site healing was addressed and the primary outcomes (CEA graft take, appearance, and pigmentation) were measured as a percentage of the transplanted area, with long-term visual evaluation using the Vancouver Scar Scale (VSS) at 2 weeks, 2 months, and 6 months post transplant [17].

**Figure 2:**
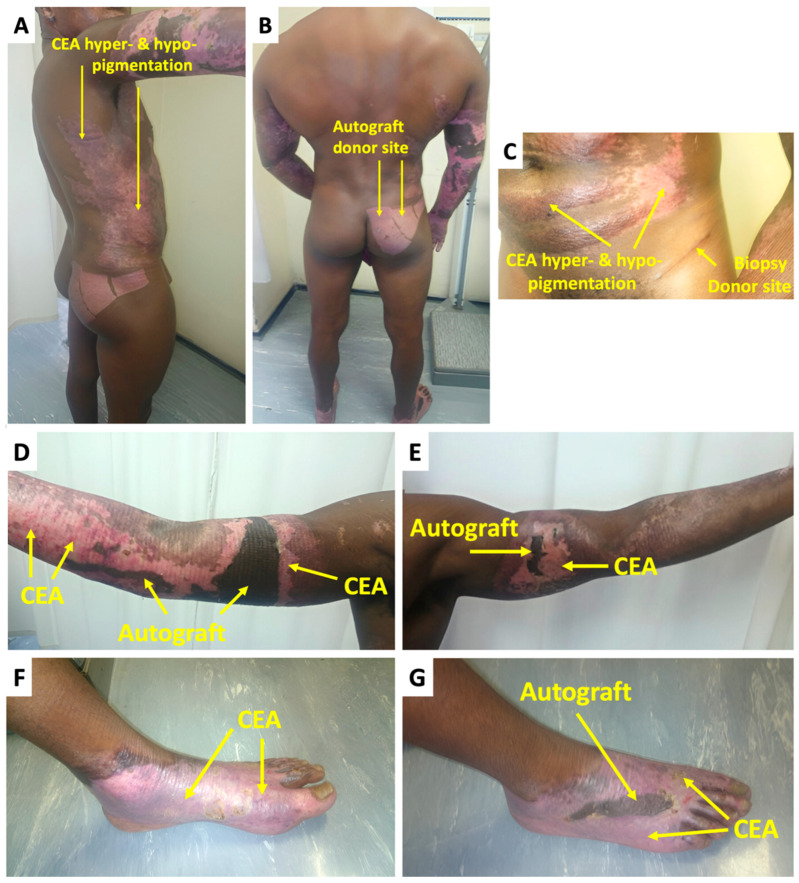
CEA and autograft after transplant during clinic follow-ups post-discharge: (**A**–**G**) CEA and autograft appearance 2 months after transplant; (**B**,**C**) autograft and biopsy donor site appearance (CEA—cultured epidermal autografts).

At week 1 after CEA transplant, there were no significant complications or indication thereof that would impede graft take (Figure 1B–D). On the final day of graft take assessment (day-21), the CEA healed well overall, with 100% graft take on the torso and left foot (Figure 1F,G). Epithelial loss and delayed healing occurred on small sections of the arms and right foot with 90 and 80% graft take (an overall collective CEA graft take of 85%), respectively. The non-healing areas were dark pink in contrast to the surrounding CEA-healed areas that were light pink (Figure 1E,H). For the non-healing areas, the patient received a small STSG of approximately 1% TBSA on the final assessment day (21 days after CEA transplant) that was harvested from the right gluteal area (Figure 1A). At the 2-month follow-up (Figure 2), the CEA demonstrated satisfactory healing by the hyper- and hypopigmentation outcomes (Figure 2A,C,F). The traditional autografts were much darker compared to even the hyperpigmented CEA areas (Figure 2D,E,G). There was less pliability in the skin graft compared to the CEA areas. The VSS assessment revealed a score of 8 at 2 weeks, while at 2 months, the score was 4 (6-month check-up revealed a VSS score = 3) (Table 1). The biopsy donor site (Figure 2C) was small with only an elongated scar remaining, which can easily blend into skin folds, whereas; although the autograft donor site (Figure 2B) could essentially easily be covered with clothing, the scars were significantly larger and more obvious.

## 3. Discussion

Eleven years ago, this modified CEA technique was first used under emergency ethical approval for a patient with prolonged hospitalization, worsening outcomes, and exhausted donor sites [13]. Since then, it has primarily been used for life-saving purposes in the absence of alternative treatments, with overall graft take rates higher [8] than the largest reported case series [9]. However, graft quality was secondary to its role in ensuring permanent coverage for survival. The high costs limit its use to severe cases, and few studies have explored CEA as a cosmetic treatment. During the course of the technique’s application at the WCPATBC, a bodybuilder with 20% TBSA and a high survival probability (98%) refused grafts from visible donor sites, citing concerns over scarring visibility affecting his profession. Since bodybuilding has reportedly been perceived as “at the very least, a subculture, whose practitioners suffer from large doses of insecurity” [18], the resultant adverse implications on appearance and insecurity have related to negative body image [19,20] and reputation regression of bodybuilding among broader audiences [21,22]. Given the emotional and professional impact of scarring in bodybuilding, CEA was chosen for its better cosmetic outcomes in terms of scar appearance [10]. The groin donor site healed very well with a minimal, concealed scar, unlike the larger scar from the right gluteal autograft. The biopsy donor site scar could easily be camouflaged in the skin fold or covered by the costumes typically worn by bodybuilders during show-casing.

The small non-healing areas that required autografting were likely attributed to shearing forces during movement or to a minor local infection that cleared without antibiotic intervention. During the immediate post-operative period (typically up to day 5, before the initial dressing change and exposure of the grafted areas), regions particularly prone to movement (such as the limbs) are immobilized using splint back slabs, especially in cases involving extensive wounds. This practice helps to minimize shear forces and optimize graft adherence. Following this initial phase, daily wound cleaning and reapplication of dressings are performed, and patients are gradually encouraged to mobilize. However, in certain instances, including the present case, splint back slabs are not employed and only bandaging is used. This approach increases patient mobility but can increase the risk of shear-related graft loss. In this patient, it is likely that this factor contributed to the partial graft loss and delayed healing observed in approximately 15% of the CEA-treated areas. However, an overall 85% graft take was achieved, and the VSS scores improved from 8 to 3 at 6 months. The CEA showed optimal pigmentation and smoother, less hypertrophic scars compared to typical autografts. The latter characteristic also observed in previous studies that successfully treated of large burn wounds [10] and extensive keloids [15] with CEA. This modified technique overcame the usual limitations of CEA [23], with good graft take, mitigated costs, and a seamless delivery system [8,24]. Long-term follow-up revealed no contracture or blistering up to 6 months, which were some of the concerns with CEA transplants in the long run [10]. Although follow-up results did not extend into year durations, the CEA used in this patient has previously demonstrated excellent stability and pliability after 8 years post-transplant in two other patients [24,25] and were previously echoed [26]. Moreover, while expected morphological changes occur during the wound remodeling phases, no major complications (such as keloid formation and skin cancers, to date) were observed in this case or among the broader patient cohort treated with this CEA [8], supporting its potential for durable, long-term graft stability. This case study highlights two key aspects: (1) the use of a cost-effective, user-friendly CEA technique and (2) its potential for expanded cosmetic applications. The excellent outcomes observed in this case suggest that the modified CEA technique could be considered more frequently for cosmetic applications. However, it is important to acknowledge that these findings are limited by the single-case, single-centre nature of the report. Further investigations involving additional cosmetic cases, direct comparisons with other reconstructive techniques and other standardized scoring systems are necessary to fully validate the versatility and broader applicability of this approach for cosmetic purposes.

## 4. Conclusions

This modified CEA technique has demonstrated its efficacy as a life-saving wound coverage treatment in burn patients, and this case study further highlights its versatility in patients with cosmetic priorities. Minimal graft-take loss, favorable pigmentation, and smoother scar appearance of both the transplanted CEA and biopsy donor site, compared to traditional autograft sites, were observed. Although a large cohort study of burned bodybuilders treated with CEA would enhance the generalizability of findings, such cases are exceedingly rare in both the literature and our tertiary burns centre’s experience. As burns predominantly affect socioeconomically disadvantaged populations, the incidence in bodybuilders—who typically maintain high levels of discipline and personal care—is understandably low. This case appears to be the first of its kind, and similar reports are likely to remain scarce due to the rarity of such presentations in clinical practice. However, alongside the previous case–cohort outcomes, these findings suggest the potential for replacing traditional skin grafting with the modified low-cost, composite CEA technique in patients whose primary objective is improved cosmesis, particularly in those for whom cosmetic outcomes are integral to their livelihood.

## 5. Patents

The skin culture technique is patented and owned by Stellenbosch University (SU, Stellenbosch, Cape Town, South Africa).

## Figures and Tables

**Table 1:** VSS criteria and scores at 2 weeks, 2 months, and 6 months after CEA transplant (VSS—Vancouver Scar Scale, CEA—cultured epidermal autografts).

VSS Criteria and Score	2 Weeks	2 Months	6 Months
Vascularity (Normal = 0, Pink = 1, Red = 2, Purple = 3)			
	2	1	1
Pigmentation (Normal = 0, Hypopigmentation = 1, Hyperpigmentation = 2)			
	2	1	1
Pliability (Normal = 0, Supple = 1, Yielding = 2, Firm = 3, Ropes = 4, Contractures = 5)			
	3	2	1
Height (Flat = 0, <2 mm = 1, 2–5 mm = 2, <5 mm = 3)			
	1	0	0

## Data Availability

The data presented in this study are available on request from the corresponding author due to the inclusion of identifiable clinical information recorded in the patient’s medical file, which is subject to confidentiality and ethical restrictions.

## Abbreviations

The following abbreviations are used in this manuscript:

CEA	Cultured epidermal autografts
STSG	Split-thickness grafts
Western Cape Provincial Adult Tertiary Burn Centre	WCPATBC
Total body surface area	TBSA
Platelet-rich plasma	PRP
Vancouver Scar Scale	VSS
